# The impact of the live broadcast of Stromae’s song *L’enfer* on social media publications, calls to the national helpline, and suicide attempt rates in France

**DOI:** 10.1192/j.eurpsy.2024.1805

**Published:** 2025-01-09

**Authors:** Charles-Edouard Notredame, Chloé Saint-Dizier, Antoine Lamer, Erika Nogue, Emile Fares, Mohamed Lemdani, Guillauma Vaiva, Philippe Courtet, Emilie Olié

**Affiliations:** 1 Department of Psychiatry, National Coordination Team of the 3114, CHU Lille, Lille, France; 2 Lille Neuroscience & Cognition (LilNCog), University of Lille, Inserm, CHU Lille, U1172, Lille, France; 3 Groupement d’étude et de prévention du suicide, Saint-Benoît, France; 4 Papageno Program, Lille, France; 5 Fédération de Recherche en Psychiatrie et Santé Mentale des Hauts-de-France (F2RSM Psy), Lille, France; 6 METRICS: Évaluation des Technologies de santé et des Pratiques médicales, Laboratoire de Biomathématiques, CHU Lille, ULR 2694, Université de Lille, Lille, France; 7 Clinical Research and Epidemiology Unit, CHU de Montpellier, University of Montpellier, Montpellier, France; 8 Institute of Functional Genomics (IGF), University of Montpellier, CNRS, INSERM, Montpellier, France; 9 Department of Emergency Psychiatry and Acute Care, Lapeyronie Hospital, CHU Montpellier, Montpellier, France

**Keywords:** helpline, social media, suicide attempt, suicide contagion, Werther effect

## Abstract

On January 9, 2022, Belgian pop singer Stromae performed his new hit “L’enfer” live on French TV. The song addresses his personal struggles with suicidal ideation. To evaluate the impact of Stromae’s performance, we modeled the evolution of hospital admission rates for suicide attempts (SAs) in France, calls to the national suicide prevention helpline (3114), and Twitter publications mentioning the singer or the helpline. We employed the Gombay test to identify change points within each time series. We identified a significant increase in mean SA rates among women aged 20–24 years 6 days after the singer’s performance. No similar effect was observed in the general population or other young age groups. The show was immediately followed by a peak in tweets referring to the singer, while Twitter activity related to the 3114 remained modest. We did not observe any increase in calls to the helpline. Celebrity testimonies about suicidal experiences can help alleviate stigma but should be accompanied by prevention messages to reduce the risk of contagion.

On January 9, 2022, the famous Belgian pop singer Stromae performed his new hit “L’enfer” (“Hell”) live on a major French evening news program, reaching over 7.3 million viewers. In “L’enfer,” Stromae explicitly discusses his experience with suicidal ideation and negative feelings, resonating with others who have faced similar struggles. The performance was perceived as a breakthrough moment in suicide prevention, promoting more open discussions about suicidal behaviors. The event also garnered significant online attention, with numerous social media posts referencing suicide prevention. However, the impact of this type of event on suicidal outcomes, help-seeking behaviors, and social media reaction remains unclear.

To evaluate the effect of Stromae’s performance, we used records from the French exhaustive discharge database, the Programme de Médecine des Systèmes d’Information (PMSI), to identify hospitalizations related to suicide attempts (SAs). We included patients aged 10 years and older, discharged between January 1, 2021, and March 31, 2023, with International Classification of Diseases codes X60–X84. Additionally, using the Tweepy API, we extracted all tweets containing the keywords “Stromae” and “3114” posted between January 1, 2021, and May 31, 2022. Finally, the IT department of 3114, the new French national helpline, provided weekly exhaustive exports of time-stamped incoming calls from its launch on October 1, 2021, to March 31, 2023. We analyzed the evolution of SA-related hospital admission rates, number of tweets, and calls to 3114 as time series, which we modeled using autoregressive integrative moving average (ARIMA) processes. To assess whether Stromae’s performance was associated with significant changes in temporal patterns, we applied a Gombay change-point test to each ARIMA model. When a significant mean change-point was identified, we split the time series, computed separate ARIMA models before and after this point, and compared the corresponding parameters. For SA time series, we first analyzed the overall population, then performed subgroup analyses on adolescents and young adults (women aged 15–19 years, men aged 15–19 years, women aged 20–24 years, and men aged 20–24 years), who are known to be at higher risk for SA, especially since the COVID-19 pandemic [1].The study protocol was approved by the Montpellier Institutional Research Board (IRB-202201299).

Between January 1, 2021, and March 31, 2023, 197,224 individuals were admitted to hospitals for SA, with a mean daily admission rate of 241 ± 33.8. Most patients were women (*n* = 152,639, 79.2%). Of the attempters, 23.0% (*n* = 35,076) were aged 15–19 years (80.5% were women), and 13.1% (*n* = 19,993) were aged 20–24 years (66.3% were women). Time series results are presented in Figure 1. The Gombay tests did not detect any significant change point in the SA time series of the general population, individuals aged 15–19 years or men aged 20–24 years around the time of Stromae’s performance. However, a significant change point was detected in the mean component of the ARIMA process for women aged 20–24 years. In this group, SA time series exhibited a breakpoint on January 15, 2022, 6 days after Stromae’s performance (*p*-value < 0.001). The estimated means shifted significantly from 14.9 (SE = 0.3) before the change point to 17.3 (SE = 0.2) after the change point, indicating an increase of 2.4 hospitalizations for SA per day. We did not detect any significant change point around Stromae’s performance in the 3114 helpline activity. A peak in tweets referencing Stromae occurred immediately after the performance, with 5,551 and 8,301 posts on January 9 and 10, 2022, respectively. Two other peaks were observed: 4,902 posts on October 15, 2021, corresponding to the album announcement, and 3,750 posts on March 4, 2022, coinciding with the album release. The maximum number of posts tagging the 3114 was 116 at the helpline’s launch, but there was no peak after Stromae’s performance. Only 44 tweets mentioned both the 3114 and Stromae the day after the performance.

**Figure 1.**
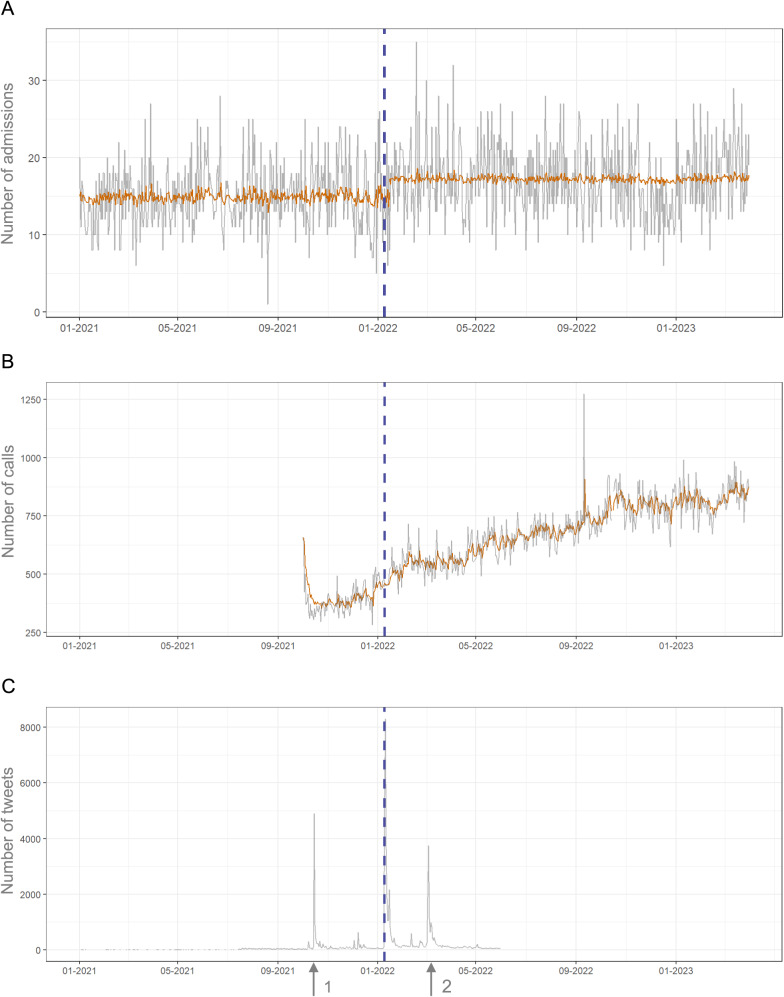
Time series analysis. A. Admissions for suicide attempt among women aged 20–24 years. The orange line represents the best fitting ARIMA predictions before and after Stromae’s intervention (purple dashed line). B. Calls to the 3114 helpline. The orange line represents the best fitting ARIMA predictions. C. Count of tweets containing a reference to Stromae. Arrow 1 corresponds to the announcement of the album release, arrow 2 corresponds to this release.

Although limited, some literature indicates that artistic performances can have either a precipitating or protective effect on suicidal behaviors. For instance, Stack et al. [2] found anecdotal evidence that the release of the song “gloomy Sunday” written by the Hungarian composer Rezső Seress triggered cases of suicides. Conversely, the broadcast of “1-800-273-8255”, a hip-hop song referring to the American National Suicide Prevention Lifeline, was associated with an increased number of calls to the helpline, as well as stronger engagements to its Twitter account [3]. The absence of concomitant increase of SA rate suggested a protective effect.

Whether media content referring to suicide provokes contagion or help-seeking behaviors, as well as the magnitude of these effects, depends on both quantitative and qualitative characteristics that are becoming better understood. First, the probability of any behavioral impact increases with the audience size of the media content [4]. Stromae’s performance took place on the most widely watched French TV show, with over 10% of the population tuning in live. The subsequent surge in social media engagement, particularly evident in increased Twitter activity, suggests a strong public interest and likely reflects the show’s wide exposure. This extensive broadcast heightened the chances that the song and its message reached susceptible viewers. Second, the impact of exposure to media content related to suicide mostly depends on its qualitative attributes. The risk of contagion appears to be particularly important for celebrity suicides [5] and sensational, dramatic, or inaccurate productions [6]. Conversely, protective effects have been associated with media content that convey hope and provide resources [6, 7]. Stromae is a globally recognized celebrity who publicly shared his mental health struggles. “L’enfer” delves into feelings of guilt and shame and reveals his suicidal ideation in straightforward poetic style that emphasizes distress. While it convokes a shared experience, it contains no message of hope for improvement, potentially conveying a sense of inevitability. During his live TV performance, the channel did not mention any helpline services like the 3114. This lack of support translated to minimal interaction on social media related to the helpline. The combination of increasing SA rates among young women and the absence of an increase in calls to the helpline may be related to a contagion effect that went unmitigated by a protective response. Third, some segments of the audience have been identified as increased risk of contagion. According to the differential identification theory, this risk is higher in individuals who admire (vertical identification) and recognize some element of themselves (horizontal identification) in the deceased celebrity [8]. Consistent with this theory, epidemiological research has consistently shown that the probability of contagion is higher in individuals of the same age and sex as the celebrity. Our results diverge with this pattern, as we found that only women younger than Stromae were affected by an increase in SA rates. However, youth is recognized to be at higher risk of suicidal contagion [9]. Under the hypothesis of the Werther effect (WE), it is possible that young women did not identify with the singer based on age or gender but connected more deeply with the emotions he expressed. Stromae’s performance coincided with the aftermath of COVID-19 pandemic, a period marked by a notable surge in suicidal ideation and SA in young women. Evidence exists that the risk of imitative behaviors would be higher in distressed individuals [10]. Therefore, “L’enfer” could have resonated most strongly within the demographic most affected by the public health crisis.

Our study is subject to several limitations: (a) The national PMSI database may underestimate the frequency of SA because it only records emergency stays lasting more than 24 hours. (b) At the time of Stromae’s performance, the 3114 had only been in operation for 4 months. The calls pattern we observed reflects a ramp-up that might have overshadowed a breaking point. (c) Due to technical constraints, we were unable to access the demographics of the individuals who called the 3114. Therefore, we cannot exclude the possibility of a specific increase in calls from sub-groups such as young women. (d) We were unable to extract the Tweet history beyond May 31, 2022, due to social media sites’ restrictions.

Because suicidal behaviors are highly multidetermined, causal assumption represents a strong and widely discussed challenge when determining the impact of media productions. While our findings do not allow us to definitively conclude that Stromae’s intervention was associated with a WE, our methodology offers solid support for this hypothesis. Interrupted time series are increasingly considered the gold standard for assessing causality in such contexts. By accounting for underlying trends and self-generated changes, they enable the detection of distinct breaks that are likely attributable to specific events. Although we cannot entirely exclude the influence of other concurrent events, the significant media impact of Stromae’s intervention suggests that it likely played a role in the break we observed in SA rates among young women. Additionally, the Gombay test, which identifies change points using a bottom-up approach, strengthens the causal argument by mitigating biases that arise from aligning time series interruptions directly with the intervention.

The present study provides a unique case study of the impact of widely publicized testimonies of suicidal experiences, particularly those involving celebrities. While such events can help reduce the stigma surrounding suicidal ideation and behaviors, they may also paradoxically have a contagious effect. Instead of censoring artistic expressions, it is crucial to foster collaboration to ensure that these testimonies are accompanied by prevention messages and resource provision.

## Data Availability

Data can be obtained by contacting the corresponding author.
